# Treatment with flaxseed oil induces apoptosis in cultured malignant cells

**DOI:** 10.1016/j.heliyon.2019.e02251

**Published:** 2019-08-09

**Authors:** Alison L. Buckner, Carly A. Buckner, Sabine Montaut, Robert M. Lafrenie

**Affiliations:** a Program in Biomolecular Sciences, Laurentian University, Sudbury, Ontario, Canada; b Health Sciences North Research Institute, Sudbury, Ontario, Canada; c Department of Chemistry and Biochemistry, Laurentian University, Sudbury, Ontario, Canada; d Northern Ontario School of Medicine, Sudbury, Ontario, Canada

**Keywords:** Cancer research, Cell biology, Cell culture, Cell death, Food science, Natural product, Nutrition, Flaxseed oil, Cancer cells, Cell death, Omega fatty acids

## Abstract

Flaxseed oil is widely recognized for its exceptional nutritional value, high concentration of fiber-based lignans and large amounts of ω-fatty acids. It is one of a generic group of functional foods that is often taken by cancer patients as a potential treatment. We have examined the anti-cancer effects of flaxseed oil by studying its direct effects on cancer cell growth *in vitro*. Treatment of a variety of cancer cell lines with flaxseed oil decreased their growth in a dose-dependent manner while non-malignant cell lines showed small increases in cell growth. Cells treated with a mixture of fatty acids, including α-linolenic acid, docosahexaenoic acid, and eicosapentaenoic acid and lignans including enterodiol and enterolactone was also able to decrease the growth of cancer cells. Treatment of B16-BL6 murine melanoma and MCF-7 breast cancer cells with flaxseed oil induced apoptosis as determined by changes in cell morphology, annexin V staining, DNA fragmentation and/or caspase activation. In addition, treatment with flaxseed oil also disrupted mitochondrial function in B16-BL6 and MCF-7 cells. These results indicate that flaxseed oil can specifically inhibit cancer cell growth and induce apoptosis in some cancer cells and suggests it has further potential in anti-cancer therapy.

## 1 Introduction

Flax (*Linum usitatissimum*), in the form of seeds or seed-derived oil, is a “functional food” recognized for its nutritional quality, high concentration of fiber-based lignans, and large amounts of ω−fatty acids [1, 2]. Flax was used to treat various health complaints in ancient Greece and Rome and was described in ancient Ayurvedic and Egyptian sources [3]. The recent popularity of flax has been bolstered by a number of studies describing health-promoting benefits such as reducing cardiovascular disease, decreasing cancer risk, inhibiting inflammatory activity, promoting gastrointestinal regularity, and alleviating menopausal symptoms [4, 5, 6]. Studies conducted in aged hens showed a diet rich in whole flaxseed decreased the risk and severity of ovarian cancer by decreasing proinflammatory prostaglandin and estrogen signaling pathways [7, 8]. The growth of estrogen-dependent MCF-7 breast cancer cells in xenotransplanted mice was also inhibited by feeding the mice flaxseed and involved decreasing hormone and growth factor signaling [9, 10]. A meta-analysis of the impact of flax on patients with breast cancer has indicated that regular consumption of flax decreased breast cancer risk, improved symptoms and survival, and was associated with improved mental health among breast cancer patients [11].

Flaxseed is one of the richest plant sources of α-linolenic acid, an ω-3 polyunsaturated fatty acid (PUFA) (49–60%), and contains a modest level of linoleic acid, an ω-6 PUFA (12–17%) [3]. It is suggested that a healthy diet should consist of a 1:4 ratio of ω-3 to ω-6 fatty acids. However, the average western diet which consists of a 1:10 to 1:30 ratio of ω-3 to ω-6 fatty acids has been linked to high levels of chronic disease and cancer [12, 13, 14]. Increasing the ω-3/ω-6 PUFA ratio has been shown to decrease cancer risk [15, 16] while increasing ω-6 PUFA has been shown to increase cancer risk [17, 18] in some human epidemiological studies, although not in all patient cohorts [6]. *In vitro* experiments and animal models suggest that diets rich in ω-3 PUFA can be protective against several cancers, such as colon or breast cancers [19, 20, 21, 22] while treatment with ω-6 fatty acids can increase cancer cell proliferation [15]. For example, mice fed diets enriched in α-linolenic acid, which increases plasma levels of α-linolenic acid and its metabolites eicosapentaenoic acid (EPA) and docosahexaenoic acid (DHA), decreases the growth of transplanted prostate, colon, and breast cancer cells [23, 24, 25, 26]. *In vitro* studies have shown that treatment of cancer cells with ω-3 fatty acids such as α-linolenic acid, DHA and/or EPA can inhibit their growth and promote apoptosis. For example, treatment of cells with α-linolenic acid can inhibit the growth and promote apoptosis of cervical, pancreatic, colon and breast cancer cells [25, 26, 27, 28, 29, 30]. In addition, treatment of colon cancer cells [31] or MCF-7 breast cancer cells [32] with α-linolenic acid, EPA or DHA was able to induce apoptosis through a mitochondrial-mediated pathway. Other experiments have shown that α-linolenic acid, DHA, and EPA can affect cell survival by altering the expression of oxidative response signaling [33], MAP kinase and NF-kB survival pathways [27], or miR-21 expression [34].

Flaxseed is also a rich source of plant lignans, such as secoisolariciresinol diglucoside (SDG), which have been shown to block cell proliferation and reduce tumor growth in experimental models possibly by modulating estrogen receptor- or growth factor-dependent signaling [9, 35]. For example, treatment of breast cancer cells with flaxseed enriched in lignans, including SDG, was able to inhibit cell growth likely by modifying estrogen signaling and downregulating the expression of ERα and ERβ [10, 19]. However, it is thought that the combination of SDG and ω-3 fatty acids is important to mediate the anti-inflammatory and anti-cancer activities [9, 16, 36].

Our experiments investigated the effects of treatment of cultured cells with flaxseed oil in order to investigate the mechanisms underlying changes in cell growth. The results indicate that treatment with flaxseed oil preferentially inhibits the growth of malignant cell cultures and were able to induce apoptosis in treated cancer cells.

## 2 Materials and methods

### 2.1 Tissue culture

B16-BL6 (murine melanoma) [37], MCF-7, MDA-MB-231, MDA-MB-468 (breast cancer), HeLa (cervical cancer), HEK293 (embryonic kidney cells) (obtained from the American Type Culture collection, ATCC, Manassas, VA), HSG (human epithelial cells [38]), and HBL-100 (breast epithelial cells [39]) (obtained from KM Yamada, NIH, Bethesda, MD) were maintained in Dulbecco's Modified Essential Medium (DMEM, Hyclone Logan UT) supplemented with 10% fetal bovine serum (Hyclone), 100 μg/ml streptomycin, and 100 U/ml penicillin (Invitrogen, Burlington, ON). The U937 and THP-1 (monocytic leukemia) (ATCC) cells were cultured in RPMI1640 medium supplemented with 10% fetal bovine serum and 100 μg/ml streptomycin, and 100 U/ml penicillin. The cells were cultured at 37 °C in 5% CO_2_. For experiments, cell cultures were treated with media containing different concentrations of flaxseed oil or sunflower oil.

### 2.2 Flaxseed oils and characterization

Flaxseed oils were obtained by extraction of flaxseeds or from commercial suppliers including Life Brand (Shoppers Drug Mart, Toronto, ON), Weber Naturals (WN Pharmaceuticals, Coquitlam BC), Swiss Natural (Valeant Pharmaceuticals, Laval, QB), and Polar Foods Inc. (Fisher Branch, MB). The Life Brand of flaxseed oil was used throughout the experiments. The sunflower oil was obtained from a commercial source. For analysis, the fatty acids were extracted and methylated according to Phippen et al. [40]. Oils were treated in 1 ml 0.5 M KOH in methanol at 60 °C for 1 h, 1 ml 1 M H_2_SO_4_ for a further 15 min, and then extracted into hexane. LC-MS analysis was performed on an Agilent G1311A/G1213A LC system and Agilent 6120 MS using a 2.1 × 250 mm Grace Smart C18, 60A, 5 μm column (Grace Discovery Sciences). The mobile phase was applied at 0.5 ml/min starting with 55% phase A (0.1% formic acid in water)/45% phase B (0.1% formic acid in acetonitrile) for 10 min and then ramped to 5% phase A/95% phase B for a further 20 min. The electrospray interface for the MS operated at 350 °C, capillary voltage was 4000V positive, 3500V negative, nitrogen gas was used at a flow rate of 10 l/min, and full scans were collected between *m/z* 100–1000. The amount of methylated fatty acids in the oils was determined from a standard curve of pure standards (FAME, Sigma-Aldrich Chemical Co., Oakville, ON) run under the same conditions.

Individual fatty acids including α-linolenic acid, docosahexaenoic acid, eicosapentaenoic acid, linoleic acid, oleic acid, and palmitic acid and the lignans enterodiol and enterolactone were purchased from Sigma-Aldrich Chemical Co. The fatty acids were solubilized in DMSO, combined in an equimolar mixture, with each component suspended in culture media at 10^−5^ M.

### 2.3 Trypan blue cell survival assay

The cell cultures (3 × 10^5^/ml) were plated on 60 mm tissue culture plates on day 0. The cells were treated with 0.3% (v/v, low concentration) or 0.9% (v/v, high concentration) flaxseed oils on day 1 and were maintained without changing the media for the duration of the experiment. Replicate plates were harvested each day for 4–6 days the cell pellets suspended in PBS, pH 7.4 containing 0.015% trypan blue, and clear (live) cells were counted on a hemocytometer. Cell counts were performed in octuplet, the numbers for each experiment averaged, and the mean ± standard deviation for fold increase reported for 3 independent experiments.

### 2.4 Methyl tetrazolium blue viability assay

The viability of the cells was measured using the methyl tetrazolium (MTT) blue assay. The cells were plated on 96-well plates at 2 × 10^3^ cells/well on day 0 and treated with the 0.9% (v/v) of flaxseed or sunflower oil on day 1 which was maintained for the duration of the experiment. Each day, 5 μl/well of a 5 mg/ml MTT solution was added to a replicate plate and after a 3 h incubation the media was removed, the cells solubilized in 100 μl DMSO, and the absorbance measured at 540 nm. Each experiment was performed using 6 wells/condition and the average determined. The percent inhibition of growth compared to cells treated only with media was determined and the mean for 3 independent experiments was reported.

### 2.5 Flow cytometry

Cell proliferation and apoptosis were measured by flow cytometry following staining with propidium iodide or annexin V. B16-BL6 and MCF-7 cells were treated with media or media containing 0.3% (v/v) or 0.9% (v/v) flaxseed or sunflower oil for 24–72 h and harvested with trypsin. For cell cycle analysis, the cells were fixed by incubation in cold 70% ethanol and then washed and incubated in 1 ml PBS, pH 7.4, containing 5 μl propidium iodide for 30 min and analyzed on a FC500 Beckman flow cytometer. For annexin V staining, the freshly harvested B16-BL6 cells were incubated in PBS, pH 7.4, containing 1% FBS for 45 min, washed, and then stained for 1 h in 1 ml PBS, pH 7.4, containing 5 μl of annexin V-FITC (Roche Diagnostics, Laval QB). The cells were then washed and analyzed on a FC500 Beckman flow cytometer. The % cells shown for each condition were calculated as the mean percent positive cells from 3 independent experiments.

### 2.6 Fluorescence microscopy – vital staining

Cell morphology was examined in cells stained with acridine orange and ethidium bromide. B16-BL6 and MCF-7 cells were plated overnight on glass coverslips and then treated with media or media containing 0.3% (v/v) or 0.9% (v/v) flaxseed or sunflower oil or 10^−6^ M camptothecin (Sigma-Aldrich Chemical Co.) as a positive control, for 24–48 h. Cells were also treated with an equimolar mixture of flaxseed oil components (fatty acids including α-linolenic acid, docosahexaenoic acid, eicosapentaenoic acid, linoleic acid, oleic acid, and palmitic acid and the lignans enterodiol and enterolactone) in culture media each at a concentration of 10^−5^ M. Cells were incubated in 10 μg/ml acridine orange and 10 μg/ml ethidium bromide for 15–30 min and visualized on a LSM5 Zeiss fluorescence microscope for apoptotic morphology. Alternately, live cell monolayers were incubated in 100 nM MitoTracker Red CMXRos (Molecular Probes, Fisher Scientific, Whitby, ON) or 100 nM Lysotracker Red DND-99 (Molecular Probes) for 15–30 min and visualized on a LSM5 Zeiss fluorescence microscope to determine the effects on organelle function.

### 2.7 Fluorescence microscopy – TUNEL

The TUNEL assay was used to detect DNA fragmentation [41]. The cells were plated on glass coverslip and then treated with media or media containing 0.3% (v/v) and 0.9% (v/v) flaxseed oil or sunflower oil. As a positive control for apoptosis, B16-BL6 cells were treated with 10^−6^ M camptothecin for 24–48 h and MCF-7 cells were treated with Ultraviolet radiation for 30 min and incubated for 24 h. The cells were fixed in 1 ml of 10% formaldehyde solution (Sigma-Aldrich Chemical Co.) for 5 min, permeabilized in 1 ml 1% Triton X-100 in PBS for 5 min and incubated with 50 μl of the TUNEL reagent (Roche Diagnostics) for 60 min at 37 °C. The coverslips were mounted on glass slides and visualized using an LSM5 Zeiss fluorescence microscope. The percent positively stained cells is shown for at least 5 independent microscope fields (>100 total cells counted/condition).

### 2.8 Immunoblot analysis

B16-BL6 or MCF-7 cells were treated with media, 0.3% (v/v) or 0.9% (v/v) flaxseed oil, and/or sunflower oil, or 10^−6^ M camptothecin for 48 h. The cells were harvested and lysed in RIPA buffer (1% Triton X-100, 0.5% SDS, 0.5% sodium deoxycholate, 150 mM sodium fluoride, 1 mM sodium orthovanadate) containing protease inhibitors (Roche Diagnostics). Total cell lysates were subjected to electrophoresis on 10% PAGE gels containing SDS and electrophoretically transferred to nitrocellulose membranes (Schleicher and Schuell, Xymotech Biosystems, Toronto, ON). The membranes were blocked by incubation in 5% BSA in Tris-buffered saline, pH 7.5 and 0.1% Tween-20 (TBST) and then incubated with antibodies against caspase-3 (#9668, Cell Signaling Technology, Danvers, MA), caspase-9 (sc-8355, Santa Cruz Biotech., Santa Cruz, CA) or PARP (sc-7150, Santa Cruz Biotech.) in 0.5% BSA in TBST. The filters were washed and incubated with appropriate anti-IgG-horseradish peroxidase conjugates (Santa Cruz Biotech.) and the HRP detected by incubation in Supersignal Reagent (Pierce Chemical Co., Rockford, IL) and exposed to Hyperfilm-ECL X-ray film (Amersham-Pharmacia, Oakville, ON). Densitometry was performed using AlfaEaseFC software (Protein Simple, San Jose, CA) and fold changes in band intensity were compared to untreated cells and reported for 3 independent experiments.

### 2.9 Caspase activity assays

The activity of caspase-2, 3, 6, 8, and 9 were assayed using the Apotarget Caspase Protease Assay kit (Invitrogen Corporation, Thermo-Fisher, Whitby, ON). B16-BL6 cells (5 × 10^6^ cells/assay) were treated with media, 0.9% (v/v) flaxseed oil or sunflower oil for 24 h, harvested using trypsin, and lysed in 50 μl of chilled Cell Lysis Buffer for 10 min. The lysates were centrifuged at 16,000 × g for 1 min and the supernatant diluted to a protein concentration of 2 mg/ml. A mixture of 50 μl of cell lysate, 50 μl of 2× reaction buffer and 5 μl of 4 mM colorimetric substrate (200 μM final concentration) was incubated at 37 °C for 1.5 h in a 96-well plate and absorbance read at 405 nm. The fold increase in caspase activity was determined by subtracting the background (no lysate control) and then dividing the flaxseed- or sunflower oil-treated samples by the media-treated controls. The results are the mean of 3 independent experiments.

### 2.10 Statistical analysis

Data were expressed as the mean ± SD of at least three independent analyses. The mean values were subjected to a one-way ANOVA followed by a Tukey *post hoc* analysis to test significant differences (*p* < 0.05) between groups. Comparison between treatment and control samples for the caspase assays were performed using a Students t-test.

## 3 Results

### 3.1 Flaxseed oil inhibits cancer cell growth

**Fig 1:**
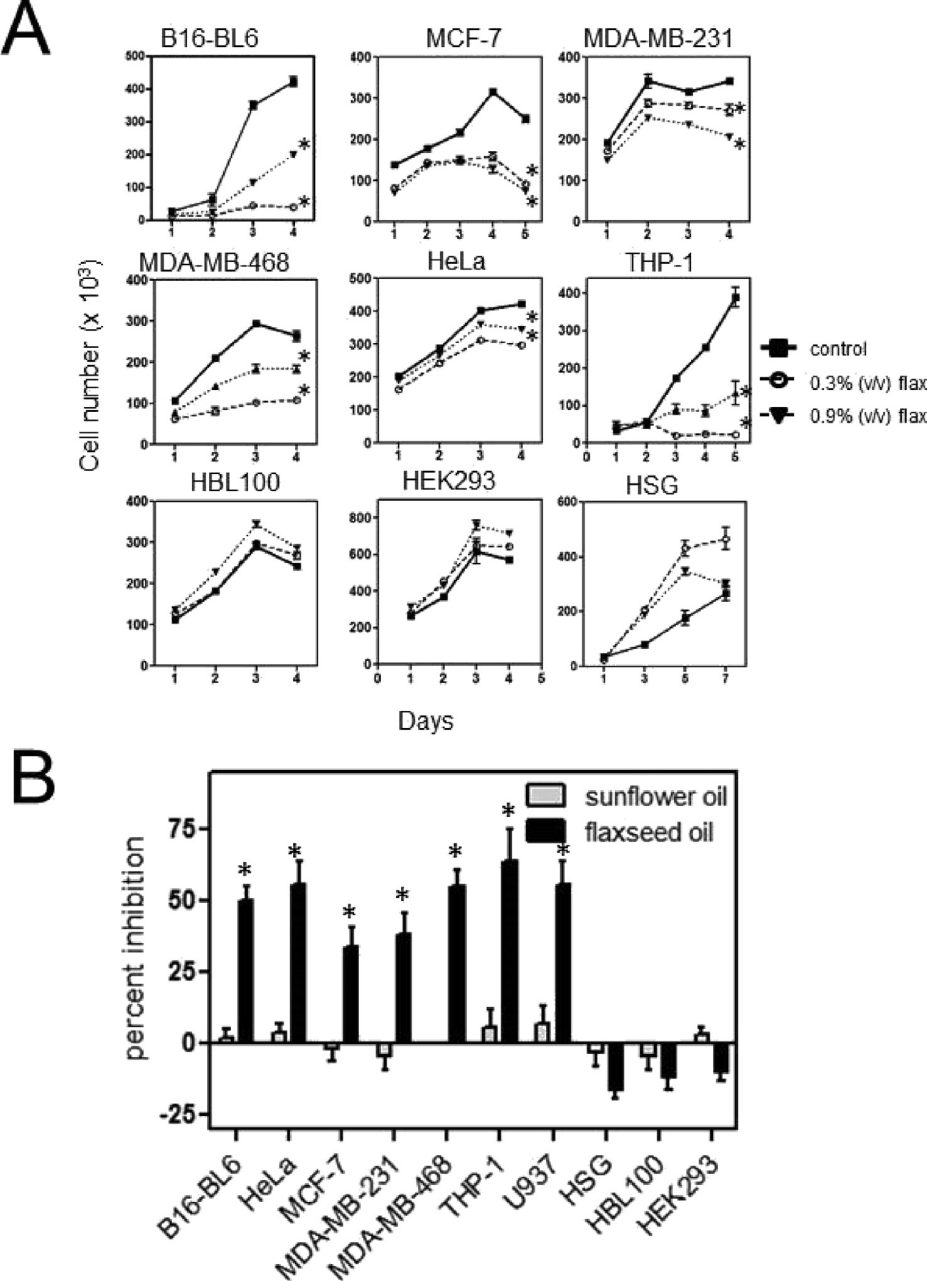
Treatment with flaxseed oil decreases proliferation of malignant cells. **A.** Malignant (B16-BL6, HeLa, MCF-7, MDA-MB-468, MDA-MB-231, and THP-1) and non-malignant (HSG, HBL100, and HEK293) cells were treated with culture media containing 0.3% or 0.9% (v/v) flaxseed oil on day 0. Replicate cultures were harvested and cell number determined each day for 4–5 days. **B.** Cells were plated on 96-well plates, treated with flaxseed oil or sunflower oil on day 0 and cell viability determined using an MTT assay. The mean ± SD percent inhibition of growth compared to media only controls for 3 independent experiments is shown for day 4 of treatment. *indicates a significant difference from the control cells (p < 0.05).

The effect of flaxseed oil on cell growth was determined for a variety of malignant and non-malignant cells lines. The cells were treated by adding different concentrations of flaxseed oil directly to the media and then the number of cells counted each day for 4–6 days. Treatment with flaxseed oil inhibited the proliferation of malignant cell lines in a dose-dependent manner. Treatment with 0.3% (v/v) flaxseed oil (low dose) for 4 days decreased the number of B16-BL6 cells by ∼50% and treatment with 0.9% (v/v) flaxseed oil (high dose) completely inhibited cell growth. Other malignant cells showed similar inhibition: 4 days after treatment with 0.3% (v/v) flaxseed oil, growth was reduced by 25% for HeLa, 50% for MDA-MB-231 and MDA-MB-468 cells, and 75% for MCF-7 cells while treatment with 0.9% (high dose) flaxseed oil reduced growth by 60% for HeLa cells, 75% for MDA-MB-231 and MDA-MB-468 cells, and 90% for MCF-7 cells (Fig. 1A). In contrast, treatment with flaxseed oil increased the number of the non-malignant human cell lines including HSG epithelial cells, HBL100 breast cells, and HEK293 embryonic kidney cells. The MTT viability assay also showed that the growth of malignant cells was inhibited by 40–60% after 4 days of treatment with flaxseed oil while non-malignant cells were not affected (Fig. 1B). In order to control for changes in the overall lipid content of the treatment media, sunflower oil was used as a control. Treating the cells with similar amounts of sunflower oil had no effect on the growth of any of the cells tested.

**Fig. 2:**
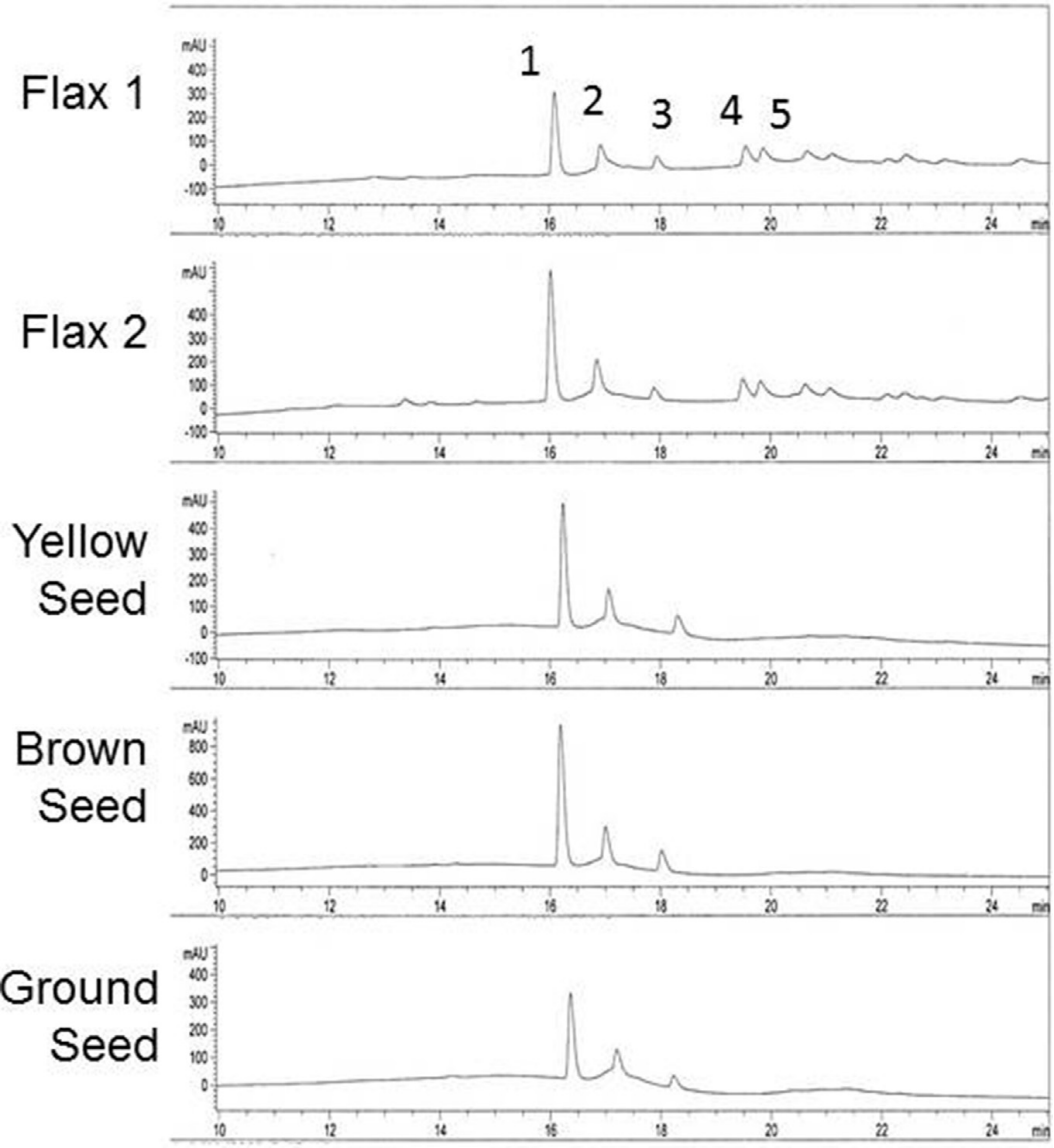
HPLC chromatograms of different oils used in these experiments. Fatty acids were extracted from flaxseed oil from different sources and methylated prior to analysis by HPLC. The identity of each peak was determined by comparison to a FAME standard. 1. α-linolenic acid; 2. linolenic acid; 3. oleic acid; 4. palmitic acid; 5. stearic acid.

Different sources of flaxseed oil had a similar composition of specific fatty acids as determined by HPLC (Fig. 2, Table 1). However, flaxseed oil from different sources had diverse effects on proliferation and blocked cell growth by 8–65% after 5 days. The flaxseed oil obtained from Life Brand had the most reproducible effects and was used in the remaining experiments. Other experiments (not shown) demonstrated that flaxseed oil lost activity after 10–14 days at 37 °C or after several months in sealed containers at room temperature.

**Table 1:** Composition and efficacy of differently analyzed flaxseed oil samples.

Oil	α-linolenic acid (mg/ml)	Linoleic acid (mg/ml)	Oleic acid (mg/ml)	Inhibition of B16-BL6 growth (%±SEM)	
				Low	High
Flaxseed oil 1	0.51	0.28	0.41	7 ± 8	13 ± 4*
Flaxseed oil 2	0.74	0.35	0.30	10 ± 9	8 ± 6
Yellow flaxseed extract	0.63	0.26	0.57	16 ± 6*	26 ± 7*
Brown flaxseed extract	1.16	0.42	1.04	34 ± 6*	28 ± 5*
Flax seed extract	0.49	0.20	0.34	28 ± 5*	53 ± 7*
Flaxseed oil (Life)	0.57	0.16	0.18	32 ± 9*	65 ± 11*
Sunflower oil	0.04	0.65	0.16	8 ± 9	4 ± 11

* p<0.05.

**Fig 3:**
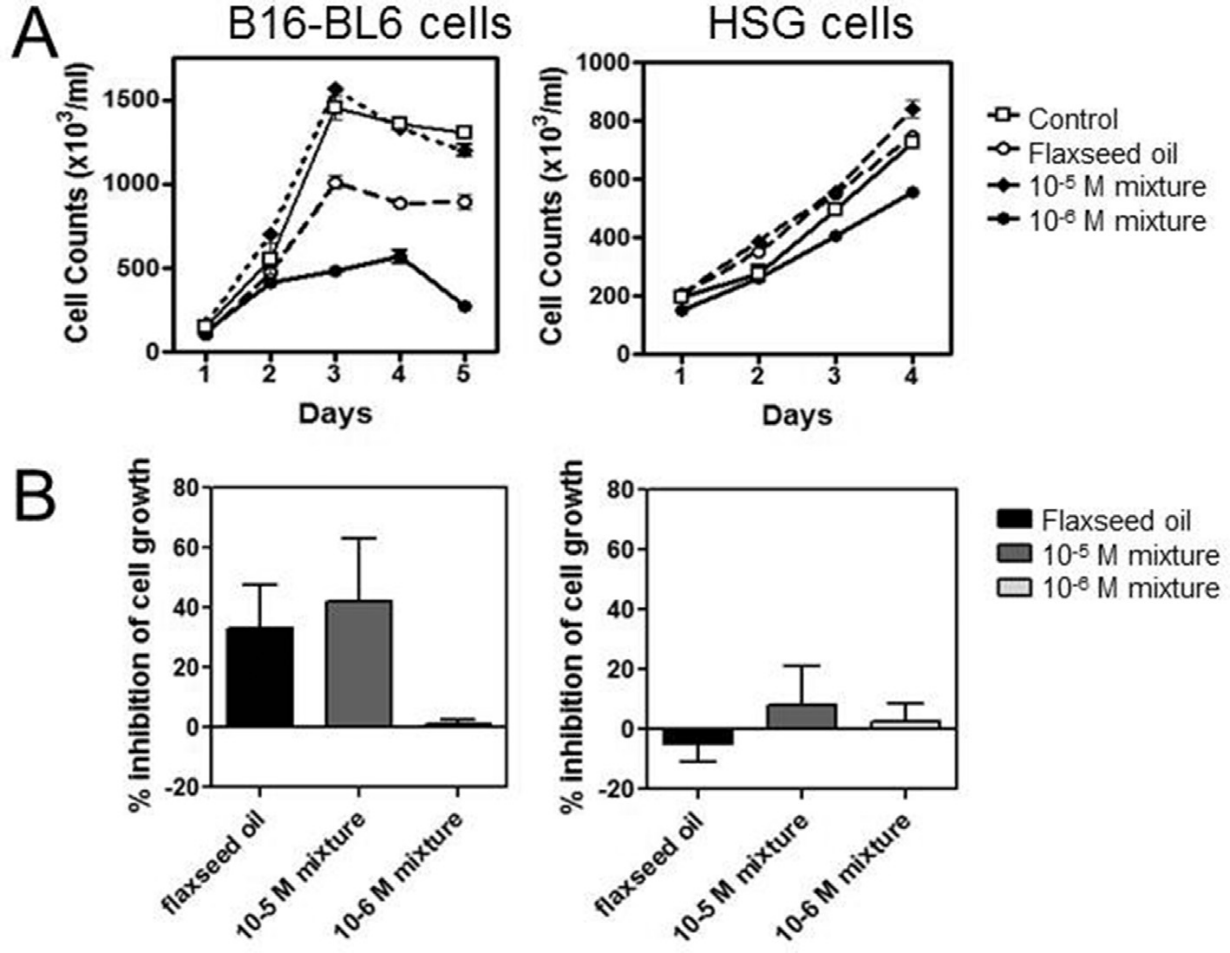
Treatment with a mixture of flaxseed oil components decreases proliferation of malignant cells. **A.** B16-BL6 and HSG cells were treated with culture media containing flaxseed oil or a mixture of fatty acids including α-linolenic acid, docosahexaenoic acid, eicosapentaenoic acid, linoleic acid, oleic acid, and palmitic acid and lignans including enterodiol and enterolactone each at 10^−6^ or 10^−5^ M. Replicate cultures were harvested and cell number was determined each day for 4–5 days. **B.** The average percent inhibition in cell proliferation compared to media only controls was determined from 3 independent experiments for cells treated for 3 days.

The proliferation of B16-BL6 and MCF-7 cells was blocked by treatment with an artificial mixture of “flaxseed” oil containing only an equimolar combination of the most common fatty acids including of α-linolenic acid, docosahexaenoic acid, eicosapentaenoic acid, linoleic acid, oleic acid, and palmitic acid, and the lignans enterodiol and enterolactone. Cells treated with the artificial mixture with each component at 10^−5^ M (or 10^−6^ M) on day 0 reduced cell growth by 45% compared to vehicle controls after 4 days (Fig. 3). Treatment of cells with the individual flaxseed oil components at the same concentrations did not affect cell proliferation (not shown).

### 3.2 Induction of apoptosis

**Fig 4:**
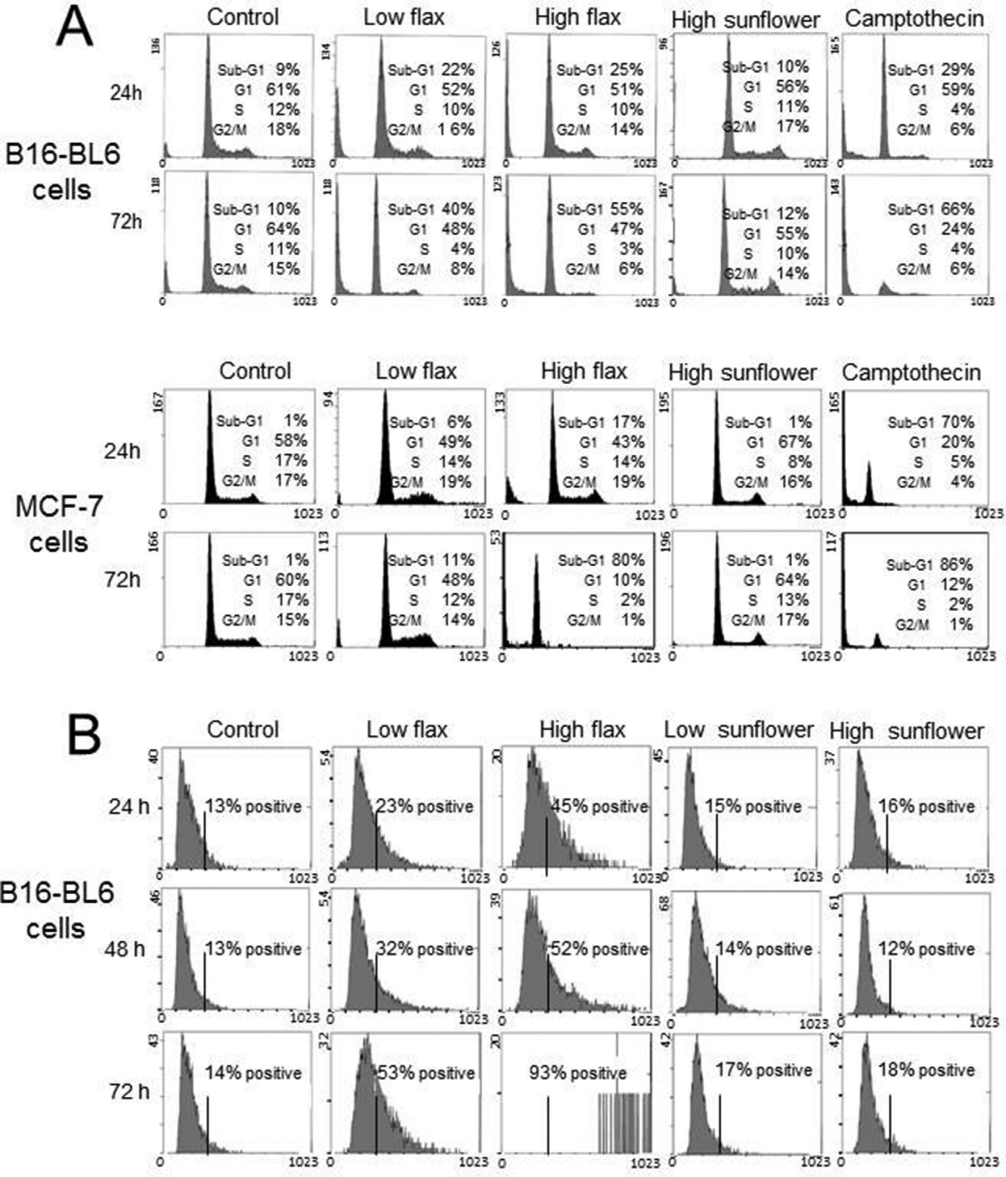
Flow cytometry of flaxseed oil treated cells shows nuclear fragmentation and annexin V binding. **A.** B16-BL6 or MCF-7 cells were treated with culture media (control), 0.3% (low flax) or 0.9% (v/v) (high flax) flaxseed oil, or 10^−6^ M camptothecin for 24 and/or 72 h, fixed in cold methanol and stained with propidium iodide. A representative DNA profile is shown and the table shows the average percent of cells in each phase for three experiments. **B.** B16-BL6 cells were treated with culture media (control), 0.3% (low) or 0.9% (v/v) (high) flaxseed oil, or sunflower oil for 24–72 h and then stained by incubation in annexin V-FITC conjugate. Cell profiles were obtained and the percent positive cells shown are the average of 3 experiments.

The effect of flaxseed oil on B16-BL6 and MCF-7 cell growth was examined by flow cytometry after propidium iodide-staining. Flow cytometry profiles for control cells treated with media showed peaks of DNA concentration corresponding to both the G1 and G2/M phases of the cell cycle (Fig. 4A) with <10% of the cells in the sub-G1 peak corresponding to cells with fragmented DNA. B16-BL6 or MCF-7 cells treated with 0.3% or 0.9% (v/v) flaxseed oil for 24 h still showed G1 and G2 peaks while the number of cells with fragmented DNA increased to 22% or 25%, respectively. After treatment with flaxseed oil for 3 days the number of cells with fragmented DNA increased to 40%–55% similar to cells treated with camptothecin and consistent with the induction of apoptosis. Treatment of B16-BL6 or MCF-7 cells with 0.9% (v/v) sunflower oil did not increase the percentage of sub-G1 cells. Treatment of B16-BL6 cells with flaxseed oil induced binding of annexin V, another marker of apoptosis. Control cells showed that only 13% of the cells bound to annexin V (Fig. 4B). Treatment with 0.3% or 0.9% (v/v) flaxseed for 48 h increased annexin V binding for 32% and 52% of the cells, respectively. Cells treated with 0.9% (v/v) flaxseed oil for 72 h showed a loss of membrane integrity and almost all cells (>90%) stained intensely with annexin V. In contrast, cells treated with 0.9% sunflower oil did not show an increase in annexin V binding and were comparable with media controls.

**Fig 5:**
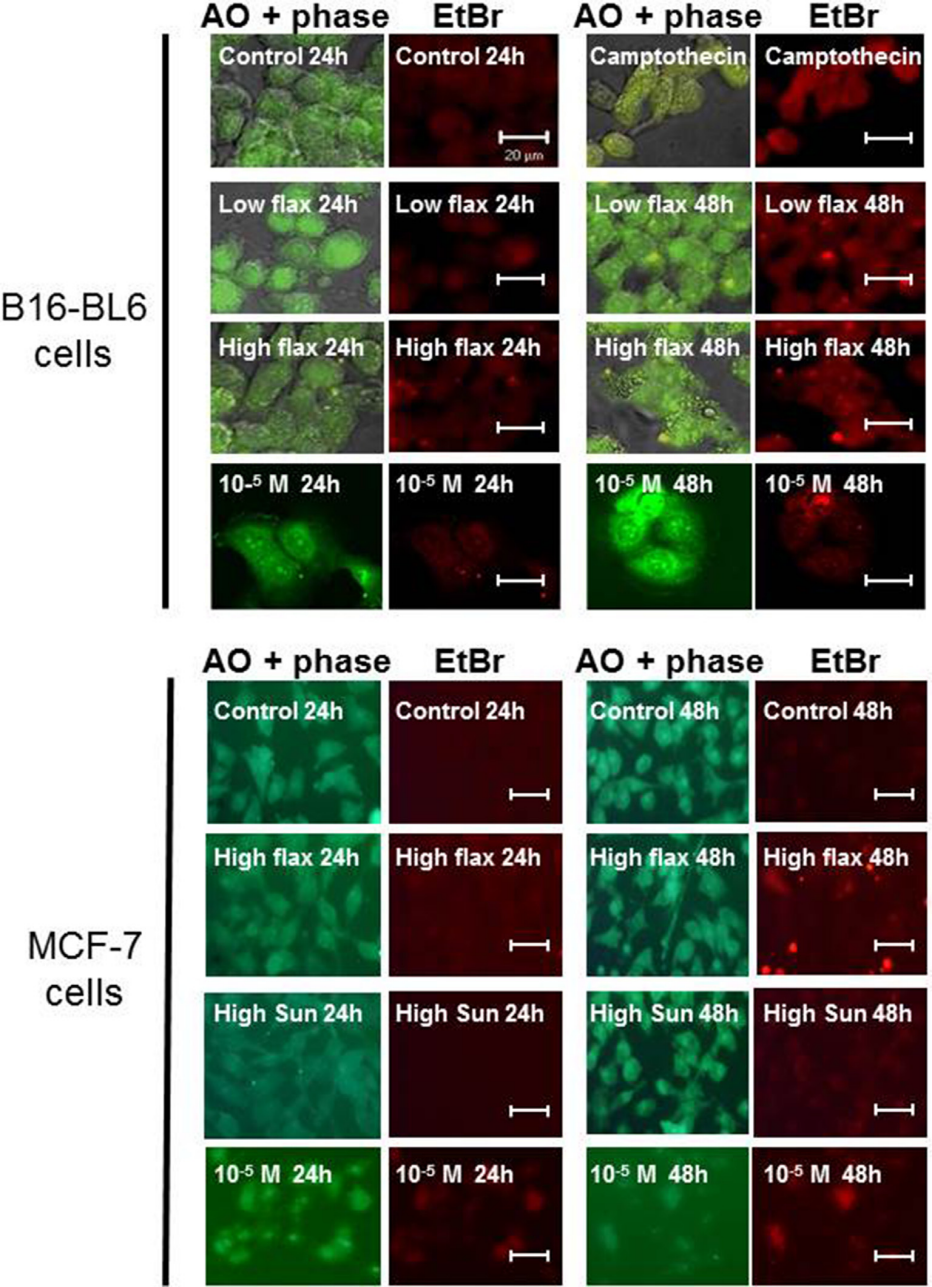
Treatment with flaxseed oil promotes apoptotic morphology. B16-BL6 or MCF-7 cells were treated with media (control), 0.3% (low flax) or 0.9% (v/v) (high flax) flaxseed oil, or 0.3% (low sun) or 0.9% (v/v) (high sun) sunflower oil or camptothecin for 24 and 48 h. Some cells were treated with a 10^−5^ M mixture of fatty acids including α-linolenic acid, docosahexaenoic acid, eicosapentaenoic acid, linoleic acid, oleic acid, and palmitic acid and the lignans enterodiol and enterolactone for 24–48 h. The cells were incubated in 10 μg/ml acridine orange (AO) and 10 μg/ml ethidium bromide (EtBr) in media for 15 min, mounted on a microscope slide and imaged on a fluorescence microscope. The bar is 20 μm.

The morphology of B16-BL6 or MCF-7 cells treated with flaxseed oil for 24–48 h was examined following staining with acridine orange and ethidium bromide. Cells treated with culture media and stained with acridine orange showed large intact nuclei (Fig. 5). Ethidium bromide did not stain the cells indicating that the plasma membrane was still intact. Cells treated with 0.3% (v/v) flaxseed oil for 24 h also showed large intact nuclei although after 48 h some of the treated cells showed areas of nuclear condensation and were stained with ethidium bromide. B16-BL6 and MCF-7 cells treated with 0.9% (v/v) flaxseed oil for 24 h showed some nuclear condensation while after 48 h the cells showed significant nuclear changes, membrane blebbing and increased ethidium bromide staining, similar to treatment with camptothecin and consistent with apoptosis. Treatment of B16-BL6 and MCF-7 cells with an equimolar mixture of the purified lipids and lignans also promoted changes in cell morphology as determined by phase contrast and fluorescence microscopy.

**Fig 6:**
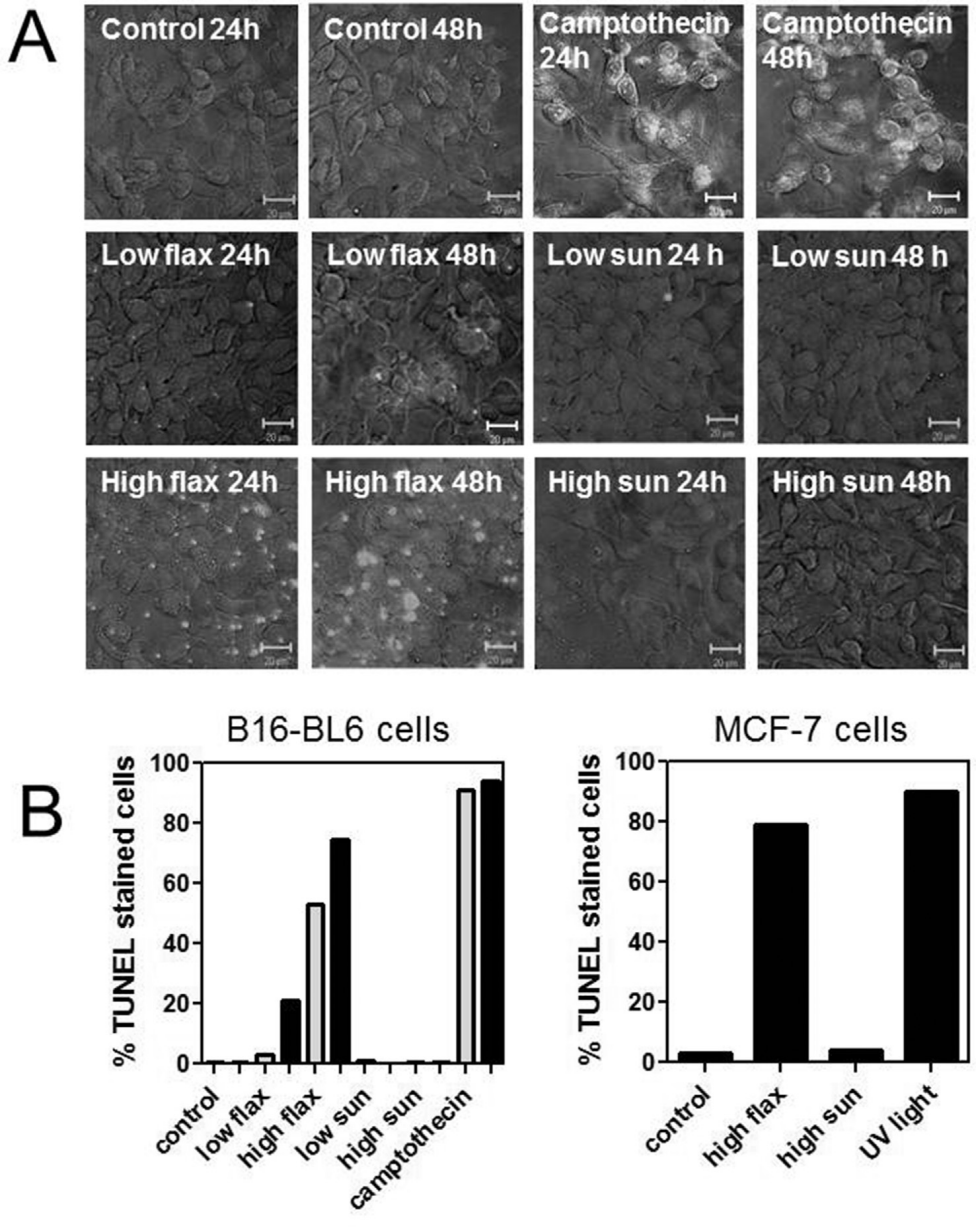
Treatment with flaxseed oil promoted DNA fragmentation *in situ*. **A.** B16-BL6 cells were treated with media (control), 0.3% (low flax) or 0.9% (v/v) (high flax) flaxseed oil, or 0.3% (low sun) or 0.9% (v/v) (high sun) sunflower oil or 10^−6^ M camptothecin for 24 and 48 h and then stained with the TUNEL reagent. Labeled cells indicate significant DNA fragmentation. **B.** The percentage of cells labeled with the TUNEL reaction mixture were determined by counting cells from at least 5 high power microscope fields (>200 cells/condition).

Treatment with flaxseed oil was also shown to promote DNA fragmentation by staining with TUNEL reagent. B16-BL6 cells treated with control media or with sunflower oil did not stain with the TUNEL reagent (Fig. 6A). While cells treated with 0.3% (v/v) flaxseed oil for 24 h did not stain, after 48 h approximately 60% of the cells were labelled. Cells treated with 0.9% (v/v) flaxseed oil for 24 h showed stained cells and after 48 h > 90% of cells showed DNA fragmentation (Fig. 6B). Similarly, treatment of MCF-7 cells with 0.9% (v/v) flaxseed oil for 48 h labeled approximately 80% of the cells while treatment with 0.9% (v/v) sunflower oil did not significantly increase TUNEL labeling.

**Fig 7:**
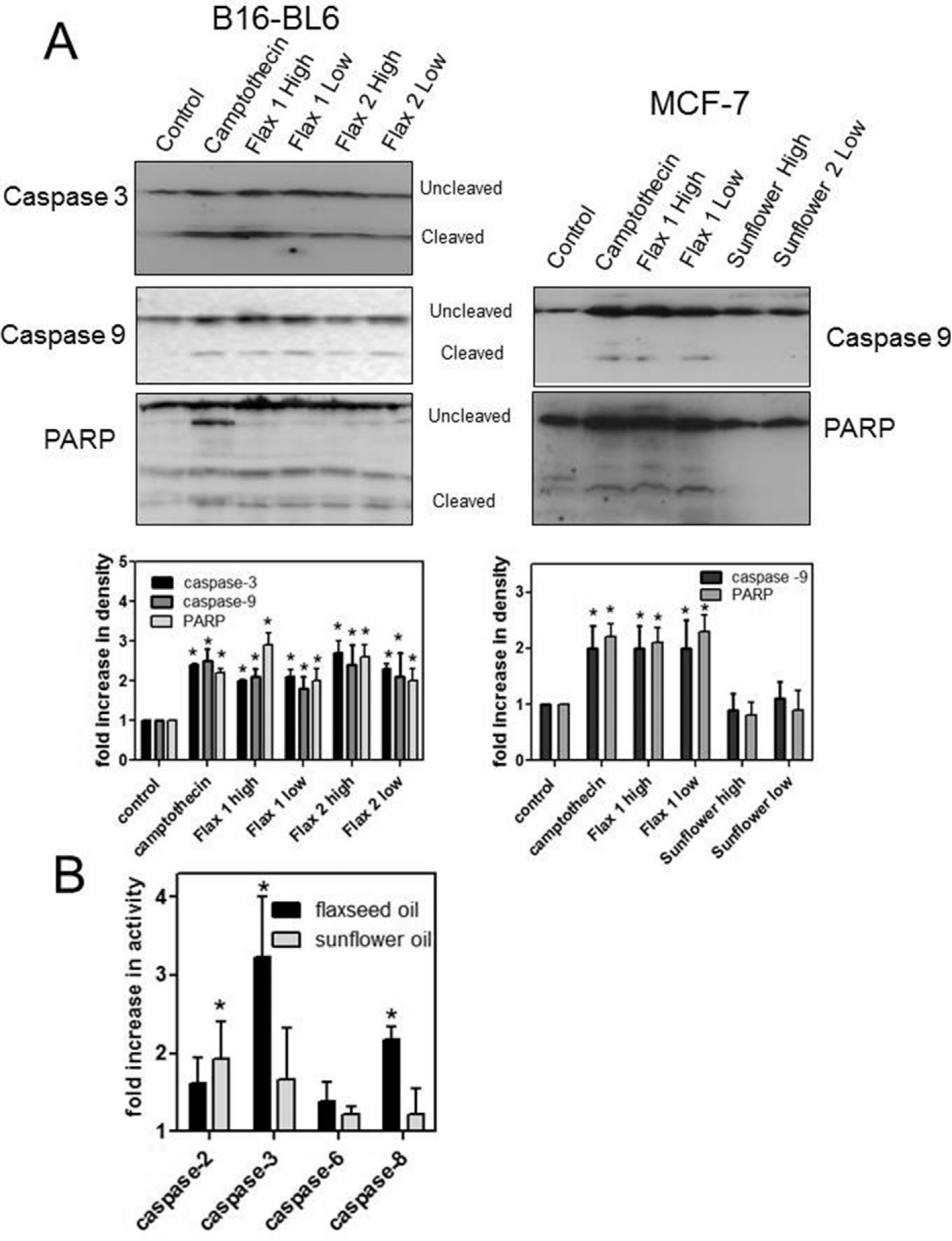
Treatment with flaxseed oil promotes activation of caspases and PARP. **A**. B16-BL6 and MCF-7 cells were treated with 0.9 or 0.3% flaxseed oil or 10^−6^ M camptothecin for 24 h and cell lysates collected and subjected to immunoblot analysis using caspase-3, caspase-9, or PARP antibodies. The relative intensities of the cleaved/uncleaved forms were determined by densitometry and the fold change for 3 independent experiments is shown. **B.** B16-BL6 cells were treated with 0.9% (v/v) flaxseed oil or sunflower oil for 24 h and then harvested and incubated with chromogenic substrates for the indicated caspases for 2 h. The relative enzyme activity was determined for each treatment by comparison to a media-treated control and is expressed as the mean ± SEM for 3 independent experiments. *indicates a significant different from the control cells (p < 0.05).

Treatment of B16-BL6 and MCF-7 cells with flaxseed oil promoted caspase and PARP cleavage and treatment of B16-BL6 cells increased caspase activity. Treatment of B16-BL6 cells with culture media showed very low levels of cleaved caspase-3, caspase-9, and PARP while treatment with flaxseed oil, at both 0.3% and 0.9% (v/v), for 24 h showed a 2–3 fold increase in the levels of the cleaved proteins that was similar to that shown by treatment with camptothecin (Fig. 7). Similarly, treatment of MCF-7 cells with flaxseed oil increased caspase-9 and PARP cleavage by 2–3 fold while treatment with sunflower oil had no effect. Treatment of B16-BL6 cells with 0.9% (v/v) flaxseed oil for 24 h also increased the activity of caspase-3 by 3.2 fold and the activity of caspase-8 by 2.2 fold compared to untreated controls (Fig. 7B). Treatment with sunflower oil increased caspase-2 activity by 1.6 fold but did not significantly affect the activity of the other tested caspases.

### 3.3 Impact on mitochondrial potential

**Fig 8:**
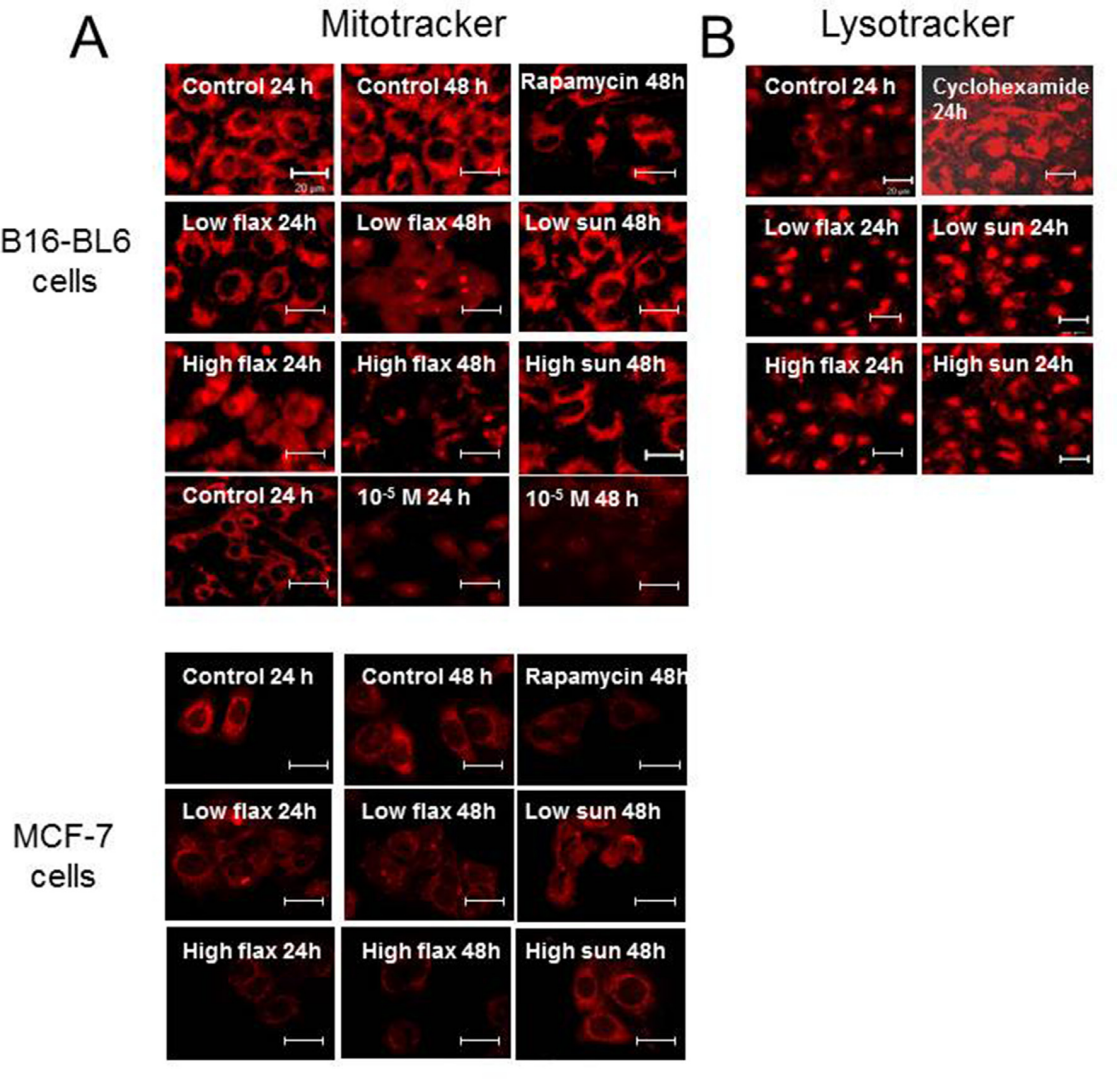
Effect of flaxseed and sunflower oil treatment on mitochondria and lysosome integrity. B16-BL6 or MCF-7 cells were treated with culture media (control), 0.3% (low flax) and 0.9% (v/v) (high flax) flaxseed oil, or 0.3% (low sun) and 0.9% (v/v) (high sun) sunflower oil for 24 or 48 h. Some cells were treated with a mixture of fatty acids including α-linolenic acid, docosahexaenoic acid, eicosapentaenoic acid, linoleic acid, oleic acid, and palmitic acid and the lignans enterodiol and enterolactone (10^−5^ M) for 24–48 h. The cells were treated with rapamycin or cyclohexamide as controls. Cells were incubated with **A.** MitoTracker-red or **B.** LysoTracker-red for 15 min and then visualized using fluorescence microscopy. The bar is 20 μm.

Treatment of B16-BL6 and MCF-7 cells with flaxseed oil also had potent effects on mitochondria membrane potential (ΔΨ) as measured using the uptake of the fluorescent dye, MitoTracker. Both B16-BL6 and MCF-7 cells normally show bright, punctate, cytoplasmic (non-nuclear) MitoTracker staining corresponding to the presence of active mitochondria (Fig. 8A). Treatment of the cells with 0.3% (v/v) flaxseed oil decreased the uptake of the MitoTracker stain after 24–48 h and disrupted the perinuclear localization of the stain. Treatment with 0.9% (v/v) flaxseed oil for 24 h decreased MitoTracker staining in both B16-BL6 and MCF-7 cells and resulted in an abnormal distribution of MitoTracker stain including in the nuclear region. Treatment for 48 h strongly inhibited mitochondrial staining similar to that shown for the rapamycin-treated cells. B16-BL6 cells treated with a mixture of the flaxseed oil components including fatty acids α-linolenic acid, docosahexaenoic acid, eicosapentaenoic acid, linoleic acid, oleic acid, and palmitic acid and the lignans enterodiol and enterolactone also promoted abnormal distribution of MitoTracker staining similar to flaxseed oil. Treatment of B16-BL6 cells with sunflower oil did not significantly alter the MitoTracker staining pattern compared to untreated cells. In contrast, the staining of lysosomes with the LysoTracker fluorescent dye was only modestly affected by flaxseed oil or sunflower oil treatment (Fig. 8B).

## 4 Discussion

The results of these studies showed that treatment with flaxseed oil can inhibit the growth of cultured malignant cells including breast cancer cells, cervical cancer cells, leukemia cells, and melanoma cells in a dose-dependent manner (Fig. 1). However, the growth of non-malignant cells was not inhibited by treatment with flaxseed oil. Since flaxseed oil preferentially kills malignant cells it may be a potential component of cancer care. Treatment of B16-BL6 mouse melanoma cells and MCF-7 breast cancer cells with flaxseed oil was able to induce apoptosis in a dose- and time-dependent manner. This is consistent with previous results which showed that treatment with flaxseed oil or ω-3 fatty acids inhibited the growth of MCF-7 and MDA-MB-231 breast cancer cells but did not affect non-malignant MCF-10A cells [19, 29, 31]. However, others have shown that both malignant and non-malignant prostate cancer cells were inhibited by ω-3 fatty acid treatment [42]. It is not clear why flaxseed oil would have a different effect on malignant versus non-malignant cultured cells or how these effects might be specific for different tissues.

Flaxseed oil obtained from different sources had variable effects on cell proliferation: some preparations were highly active at inhibiting cell proliferation while other preparations had no effect. Analysis of the major fatty acids peaks in the differently sourced flaxseed oils showed similar levels of the major components including α-linolenic acid, linoleic acid, and oleic acid which indicate the differences between oils were not likely due simply to changes in the levels of the measured major fatty acid components. The levels of α-linolenic acid were characteristically high in all of the flaxseed oil samples (0.5–1.2 mg/ml) (Table 1) comprising 40–60% of lipid while the levels of linoleic acid (0.2–0.4 mg/ml) or oleic acid (0.3–0.6 mg/ml) each comprised less than 20% of fatty acid. Treatment of cells with a combination of individual fatty acids including α-linolenic acid, docosahexaenoic acid, eicosapentaenoic acid, linoleic acid, oleic acid, and palmitic acid and the lignans enterodiol and enterolactone was sufficient to inhibit cell proliferation although treatment with an equivalent concentration of each individual fatty acid did not have an effect. This suggests that cell death induced by flaxseed oil may require collaboration among several fatty acid and/or lignan components. Further studies are required to identify specific “active factors” in flaxseed oil. However, exposure to air for over 1 week or heating [43] which causes fatty acid oxidation was shown to decrease efficacy and the differences between different sources of flaxseed oil might be related to storage and oxidation of the major fatty acids.

Adding the flaxseed oil directly to the serum-containing culture media was sufficient to induce the changes in cell proliferation. To address the potential impact of a separate oil “layer” on the cultured cells the addition of similar amounts of sunflower oil was used as a control treatment. Sunflower oil has a significantly different fatty acid (and lignan) profile from flaxseed oil and contains relatively low levels of α-linolenic acid and much higher levels of linoleic acid [44]. Treatment with the same amount of sunflower oil did not alter the proliferation or increase apoptosis in any of the cultured cell lines suggesting the mechanism of flaxseed action was not simply related to the presence of an oil but rather that the composition of the flaxseed oil was important in promoting its effect.

Treatment of B16-BL6 mouse melanoma and MCF-7 breast cancer cells with flaxseed oil did not directly block the cell cycle since propidium iodide staining showed the presence of cells in the S and G2/M phases even after 24 h of treatment (Fig. 4). However, there was a significant increase in the sub-G1 population in treated cells as early as 24 h after treatment indicating the activation of nuclear fragmentation which is consistent with apoptosis. In contrast, other studies have reported that prostate cancer cells treated with ω-3 fatty acids arrest in G2/M of the cell cycle before undergoing apoptosis [45]. Treatment of B16-BL6 cells with flaxseed oil also induced a significant increase in annexin V binding which is associated with phosphatidyl serine “flipping” to the outer leaflet of the plasma membrane and is an early marker of apoptosis [46]. More than 90% of cells treated with a high dose (0.9% v/v) of flaxseed oil for 3 days bound annexin V. This is consistent with other studies that have shown increased annexin V binding in cultured breast, prostate, or colon cancer cells treated with flaxseed oil or α-linolenic acid [17, 25, 30, 31].

Changes in cell morphology, following acridine orange/ethidium staining, and/or increased DNA fragmentation, as shown by TUNEL staining, showed that treatment with flaxseed oil promoted apoptosis of B16-BL6 and MCF-7 cells (Fig. 6). Acridine orange staining of cells [47] treated with flaxseed oil, or with a combination of lipids and lignans, for 48 h showed nuclear condensation and blebbing of cell membranes followed by a loss in plasma membrane integrity marked by staining with ethidium bromide. Treatment of B16-BL6 and MCF-7 cells with flaxseed oil also increased caspase and PARP cleavage consistent with activation of apoptosis (Fig. 7). The activation of caspase-9 is consistent with activation of the intrinsic apoptotic pathway that results from permeabilization of the mitochondria and activation of the apoptosome and ultimately results in activation of the “execution” caspase-3 [48]. Treatment of B16-BL6 cells with flaxseed oil also increased caspase-3 and caspase-8 activity which is also consistent with apoptosis. The increase in caspase-3 cleavage and increased caspase-3 activation in B16-BL6 cells treated with flaxseed oil for 24 h is consistent with previous studies in cancer cells treated with flaxseed oil or α-linolenic acid [19, 25, 31, 45]. Further, the activation of caspase-8 in flaxseed oil-treated cells is consistent with some previous observations [49]. Caspase-8 is associated with the extrinsic apoptosis pathway that results from a receptor-mediated activation of an apoptotic signal in the plasma membrane [48]. These results suggest the possibility that treatment with flaxseed oil can activate both the intrinsic and extrinsic apoptotic pathways although it is possible that caspase-3 is activated by only one pathway and then subsequently cleaves additional caspases. Interestingly, treatment with sunflower oil caused a small increase in caspase-2 activity but did not activate caspases-3, 6, 8, or 9. It is unclear whether the increased activity of caspase-2 is transient in these cells since treatment with sunflower oil does not activate any other apoptotic markers.

Our results also showed that treatment with flaxseed oil or a combination of individual fatty acids including α-linolenic acid, docosahexaenoic acid, eicosapentaenoic acid, linoleic acid, oleic acid, and palmitic acid and the lignans enterodiol and enterolactone decreased labelling of mitochondria with MitoTracker Red which is consistent with a collapse in mitochondrial membrane potential (Δψ) as shown by others in response to flaxseed or α-linolenic acid treatment [31] (Fig. 7). Treatment with sunflower oil did not affect mitochondria labelling and lysosomal labelling with Lysotracker Red was not significantly blocked by either flaxseed or sunflower oil. The effect of flaxseed oil on mitochondrial function would be consistent with activation of endogenous apoptotic pathways although further studies are required to demonstrate this linkage.

In our studies, the cells were treated directly with the flaxseed oil and this was able to inhibit cell proliferation and promote apoptosis. Many studies use incorporation of flaxseed (or PUFA) into the diet of experimental animals or humans to show an impact on cancer cell growth (or other health measures). In mouse models, changes in cancer cell growth are measured as decreases in tumour cross-sectional area or weight in response to a 5–10% flax diet [9, 35, 39]. Clinical studies with human patients that have shown health benefits from flaxseed oil also involve dietary intake of the flaxseed [11, 12, 15, 50]. The *in vivo* experiments involve absorption and metabolism of the flaxseed from diet which likely has consequences on the relative amounts of the flax components that are present in the tumour (or tissue) environment to cause the anti-tumour effects. Some studies have shown that animals or humans fed diets with a high level of flaxseed (or α-linolenic acid) can cause significant changes in the membrane lipids in tissues. For example, lipid fractions isolated from tissues or blood, including purified erythrocytes [44, 51] show incorporation of ω-3 related lipids including α-linolenic acid, and the long-chain metabolites eicosatetraenoic acid, eicosapentaenoic acid, and/or docosapentaenoic acid [52, 53]. The fact that direct presentation of the oil in media was able to affect cell proliferation suggests either that the oil contains the specific dietary active component or that the cancer cells themselves are able to metabolize the oil to create the active agent. Studies to examine the impact of flaxseed oil treatment of cultured cells on membrane lipid composition are underway.

## Declarations

### Author contribution statement

Alison Buckner: Conceived and designed the experiments; Performed the experiments; Analyzed and interpreted the data; Wrote the paper.

Carly Buckner: Performed the experiments; Analyzed and interpreted the data; Wrote the paper.

Sabine Montaut: Analyzed and interpreted the data; Contributed reagents, materials, analysis tools or data; Wrote the paper.

Robert Lafreni: Conceived and designed the experiments; Performed the experiments; Analyzed and interpreted the data; Contributed reagents, materials, analysis tools or data; Wrote the paper.

### Funding statement

This work was supported by Northern Cancer Foundation, Sudbury, Ontario; Cancer Therapeutics Research Initiative, Sudbury, Ontario; National Sciences and Engineering Research Council of Canada (NSERC Research Tools and Instruments), and Canadian Foundation for Innovation-Ontario Research Fund, Laurentian University.

### Competing interest statement

The authors declare no conflict of interest.

### Additional information

No additional information is available for this paper.
